# Loss of PTH 1 receptor signaling in periodontal cells drives cementum dysfunction and molar ankylosis in mice

**DOI:** 10.1038/s41413-026-00533-5

**Published:** 2026-04-27

**Authors:** Hakan Turkkahraman, Carson Joseph Walton, Tianli Zhu, Nisreen Akel, Silvia Marino, Xue Yuan, Teresita M. Bellido

**Affiliations:** 1 Department of Orthodontics and Oral Facial Genetics, Indiana University School of Dentistry, Indianapolis, IN USA; 2 Department of Otolaryngology-Head & Neck Surgery, Indiana University School of Medicine, Indianapolis, IN USA; 3 Department of Biomedical Sciences and Comprehensive Care, Indiana University School of Dentistry, Indianapolis, IN USA; 4 Department of Physiology and Cell Biology, University of Arkansas for Medical Sciences, Little Rock, AR USA; 5 Indiana Center for Musculoskeletal Health, Indiana University School of Medicine, Indianapolis, IN USA; 6 Central Arkansas Veterans Healthcare System, John L. McClellan, Little Rock, AR USA

**Keywords:** Bone, Bone quality and biomechanics

## Abstract

Parathyroid hormone 1 receptor (PTH1R) signaling is critical for mineral ion homeostasis and skeletal development. Although its role in tooth root formation and eruption is established, its specific functions in adult periodontal tissues and craniofacial integrity remain incompletely defined. Here, we investigated the craniofacial and dentoalveolar phenotypes of mice with conditional deletion of PTH1R in *DMP1-Cre*-expressing cells. *DMP1-Cre;PTH1R*^*fl/fl*^ mutant mice exhibited craniofacial alterations, including reduced maxillary length and defects in the alveolar bone surrounding the molars, as revealed by micro-computed tomography and histological analysis. The mutant mice also displayed severe periodontal ligament (PDL) loss and extensive molar ankylosis, characterized by the direct fusion of alveolar bone to tooth roots, predominantly in regions of acellular cementum. In contrast, incisor development remained unaffected. PTH1R deficiency also resulted in pathological cementum overgrowth, disrupted PDL fiber organization, and decreased expression of key PDL matrix proteins, as evidenced by immunohistochemical and molecular analyses. Mechanistically, the loss of PTH1R enhanced Smad3 phosphorylation and upregulated Osterix, thereby promoting aberrant cementoblast differentiation and mineralization. Concurrently, Dkk1 expression was increased, leading to suppressed Wnt signaling. This evidence establishes PTH1R signaling in cementocytes as a central safeguard of cementum homeostasis and PDL integrity and demonstrates that its disruption induces pathological root-bone fusion and craniofacial abnormalities. These findings advance our understanding of the molecular mechanisms underlying adult periodontal tissue maintenance and open new opportunities for developing therapeutic strategies against ankylosis and related disorders by targeting PTH1R signaling.

## Introduction

Parathyroid hormone 1 receptor (PTH1R) is a G protein-coupled receptor that mediates the actions of parathyroid hormone (PTH) and parathyroid hormone-related protein (PTHrP), and plays a critical role in maintaining mineral ion homeostasis.^1–3^ In the axial and appendicular skeletons, PTH1R governs bone remodeling and development through its regulation of osteoblast and osteoclast activity.^1,4^ However, less is known about its role in the bones and other tissues of the oral cavity.

The periodontium is a specialized tissue complex that anchors the tooth to the bone and comprises mineralized and non-mineralized tissues, including the cementum, the alveolar bone, gingiva and the periodontal ligament (PDL). Within the periodontium, PTH1R is expressed in cementoblasts, PDL cells, as well as in alveolar bone osteoblasts and osteocytes.^5^ Although mutations in the PTH1R gene are primarily associated with primary failure of tooth eruption (PFE) in humans,^6,7^ recent evidence indicates that affected individuals may present a wider range of dental phenotypes. These features include significant root dysplasia manifesting as severely shortened or malformed roots that compromise tooth stability and reduce the likelihood of long-term tooth retention.^7,8^ In addition to root dysplasia, alveolar bone hypoplasia is a prominent feature in affected individuals, characterized by underdevelopment of the alveolar process that impairs both function and esthetics.^9,10^ Affected patients also commonly exhibit a predisposition to dental ankylosis, a condition where teeth become fused to surrounding alveolar bone, preventing both physiological and orthodontically assisted movement.^7,11^ The phenotypic spectrum further includes specific cementum abnormalities affecting the PDL apparatus, as PTH1R mutations disrupt acellular cementum formation crucial for PDL attachment.^8,10^ Notably, these mutations can affect both primary and permanent dentitions, as evidenced by infra-occluded primary molars observed in patients with confirmed PTH1R mutations.^6^ The severity and distribution of these abnormalities vary significantly, with some patients exhibiting unilateral manifestations while others presenting bilateral involvement. Some individuals also show isolated posterior teeth abnormalities, whereas others demonstrate more widespread effects throughout the dentition process.^6^ The diverse array of dental phenotypes reflects the essential role of PTH1R during multiple stages of tooth development and may be mediated through actions on different target cells of the periodontium.

Global PTH1R knockout mice exhibit embryonic lethality due to severe skeletal mineralization defects,^12^ thus precluding their use for postnatal phenotypic analysis. To address this limitation, conditional mouse models using lineage-specific Cre drivers have been developed. *Prx1-Cre;PTH1R*^*fl/fl*^ mice, which target limb and craniofacial mesenchymal progenitors, display arrested tooth eruption, reduced alveolar bone volume, and disorganized PDL due to impaired osteogenic differentiation.^13,14^ Similarly, *Osterix-Cre;PTH1R*^*fl/fl*^ mice, which target all mesenchymal progenitors, exhibit PFE and profound root truncation.^15^ Disrupting PTH1R signaling in PTHrP-expressing dental follicle progenitors impairs normal acellular cementum formation and PDL differentiation, leading to root defects.^16^ In contrast, gain-of-function mutations in PTH1R produce distinct dental and skeletal phenotypes. For example, mice expressing Jansen-type *PTH1R*^*H223R*^ mutants that exhibit constitutive receptor activation display delayed dentin mineralization and osteoporotic alveolar bone.^17^

In this study, we investigated the specific contribution of PTH1R signaling within mature, resident osteocytes and cementocytes to the maintenance and homeostasis of the adult periodontium, particularly in preventing pathological conditions like ankylosis that may arise post-developmentally. We examined the craniofacial and dentoalveolar effects of conditionally deleting PTH1R in alveolar bone osteocytes and cementocytes, the terminally differentiated cells embedded in the extracellular matrix, using *DMP1-Cre* mice. Loss of PTH1R in these cells caused severe PDL loss and extensive molar ankylosis, primarily due to pathological cementum overgrowth. Mechanistically, PTH1R deficiency led to increased Smad3 phosphorylation and upregulation of Osterix, as well as increased Dkk1 expression and decreased Wnt signaling, driving abnormal cementoblast differentiation and mineralization. Our findings identify alveolar bone osteocytes and cementocytes as essential targets of PTH1R signaling for maintaining cementum homeostasis and PDL integrity and demonstrate that disruption of this pathway directly causes pathological tooth-bone fusion and craniofacial abnormalities.

## Results

### Craniofacial and dentoalveolar phenotypes in *DMP1-Cre;PTH1R*^*fl/fl*^ mutant mice

To investigate the role of PTH1R in craniofacial and dentoalveolar development, we conducted a detailed phenotypic analysis of *DMP1-Cre;PTH1R*^*fl/fl*^ mice. 3D μCT reconstructions revealed that, compared to control mice, mutant mice exhibited significantly reduced premaxillary and maxillary lengths (Fig. S1A, B, quantified in C). *DMP1-Cre;PTH1R*^*fl/fl*^ mice also displayed a reduction in midface length, while palatal length remained unchanged (Fig. S1A, B, quantified in C). Significant reductions were also observed in transverse dimensions of the midface in mutant mice (Fig. S1D, E, quantified in F). Intermolar, maxillary, and palatal widths were all significantly narrower in mutants compared to controls, while no significant change was found in the premaxillary width (Fig. S1D, E, quantified in F). Transverse sections of the maxillary molar region further confirmed these morphological changes, revealing abnormalities in the alveolar bone region surrounding the molars (Fig. S1G, H). In contrast, analysis of the mandible revealed no significant differences in mandibular length, intermolar width, or alveolar width between mutant and control mice (Fig. S1I–L, quantified in M).

**Fig. 1:**
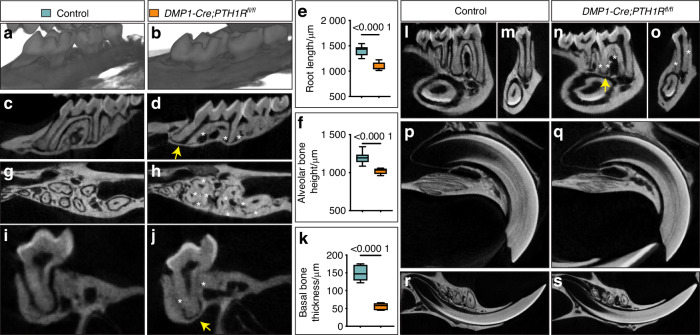
µCT characterization of molar ankylosis and basal bone defects in *DMP1-Cre;PTH1R*^*fl/fl*^ mice. **a**, **b** Representative 3D µCT reconstruction images of maxillary molars. **c**, **d** Representative sagittal 2D μCT sections of maxillary molars. **e** Quantification of maxillary first molar mesial root length (*n* = 8). **f** Quantification of alveolar bone height in the maxillary first molar furcation area (*n* = 8). **g**, **h** Representative transverse 2D μCT sections of maxillary molars. **i**, **j** Representative sagittal 2D μCT sections of maxillary first molar distal roots and surrounding alveolar bone. **k** Quantification of the basal bone thickness beneath the maxillary first molar distal roots (*n* = 8). **l**, **n** Representative sagittal 2D μCT sections of mandibular molars. **m**, **o** Representative coronal 2D μCT sections of mandibular first molar distal roots. **p**, **q** Representative sagittal 2D μCT sections of maxillary incisors. **r**, **s** Representative sagittal 2D μCT sections of mandibular incisors. Data are expressed as the mean ± SD. Asterisks indicate ankylosis regions; arrows indicate areas of thin basal bone

Next, we examined the maxillary and mandibular dentoalveolar structures. 3D reconstructions revealed no overt differences in eruption status between *DMP1-Cre;PTH1R*^*fl/fl*^ mutants and controls (Fig. 1a, b). However, sagittal μCT sections demonstrated that although mutant molars had successfully erupted, root length was significantly reduced compared to controls (Fig. 1c, d, quantified in e). Notably, the reduction in root length occurred in the context of a markedly decreased alveolar bone height (Fig. 1f). In addition, ankylosed regions—where the root surface was fused directly to the alveolar bone—were detected in all three anatomical planes of mutant mice (asterisks, Fig. 1d, h, j), which were absent in the corresponding control sections (Fig. 1c, g, i). Additionally, thinning of the basal bone underlying the molar roots was frequently observed in *DMP1-Cre;PTH1R*^*fl/fl*^ mutants, as indicated by yellow arrows (Fig. 1d, j). Quantitative analysis confirmed a significant reduction in basal bone thickness beneath the maxillary first molar distal roots in mutants compared to controls (Fig. 1k). In the mandible, similar defects were present, including abnormal ankylosis and pronounced thinning of the surrounding basal bone (yellow arrows, Fig. 1l–o). By contrast, in both the maxilla and mandible, incisors in *DMP1-Cre;PTH1R*^*fl/fl*^ mutant mice displayed normal morphology with no evidence of ankylosis or other structural abnormalities (Fig. 1p–s).

Additionally, morphometric analysis of dentin thickness revealed no significant differences between *DMP1-Cre;PTH1R*^*fl/fl*^ mice and control littermates (Fig. S2A, B, quantified in C). Histological examination of sagittal sections demonstrated that the cellular organization of the odontoblast layer and dentin matrix remained intact in the mutant mice (Fig. S2D, E), indicating that PTH1R deficiency does not substantially affect dentin formation or structure.

Collectively, these results indicate significant aberrations in dentoalveolar structures specifically in the posterior regions, while anterior structures remain unaffected. These aberrations include severe ankylosis in molars, reduced alveolar bone heights, and thinned cortical basal bone around the apices.

### Periodontal disruption and ankylosis in *DMP1-Cre;PTH1R*^*fl/fl*^ mutant mice

**Fig. 2:**
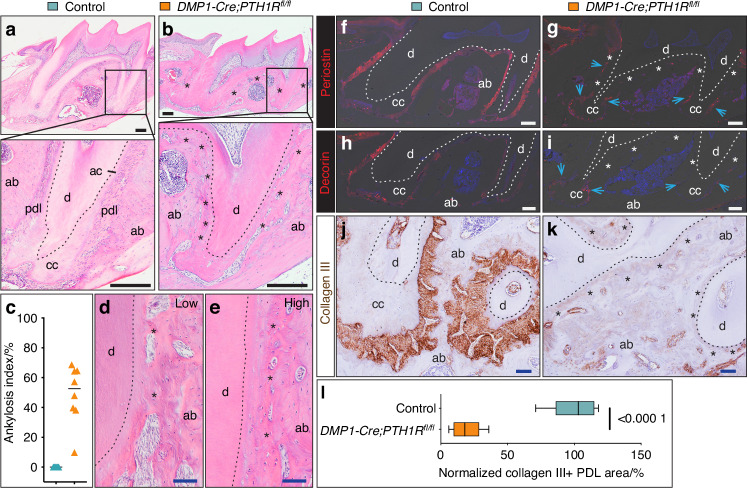
Severe PDL loss in *DMP1-Cre;PTH1R*^*fl/fl*^ mice. **a**, **b** Representative H&E-stained sagittal sections of maxillary molars. Insets show enlarged views of the distal root of the first molar, highlighting the intact PDL in controls and its absence in mutants, with direct fusion of alveolar bone to dentin (asterisks). **c** Quantification of ankylosis index (%) (*n* = 8). **d**, **e** H&E-stained sections demonstrating varying degrees of ankylosis severity in *DMP1-Cre;PTH1R*^*fl/fl*^ mutant molars, with (**d**) low and (**e**) high ankylosis severity. **f**, **g** Immunofluorescence staining for Periostin. **h**, **i** Immunofluorescence staining for Decorin. **j**, **k** Immunohistochemical staining of Collagen III. **l** Quantitative analysis of Collagen III-positive area normalized to the total tooth area (*n* = 8). Data are expressed as the mean ± SD. Dotted lines mark the dentin boundary. Abbreviations: ab, alveolar bone; ac, acellular cementum; cc, cellular cementum; d, dentin; pdl, periodontal ligament. Asterisks indicate ankylosis regions; arrows indicate remaining PDL. Scale bars: 100 µm (black) and 50 µm (blue)

To investigate the impact of PTH1R signaling on periodontal attachment structures, we performed histological analysis of molar regions in *DMP1-Cre;PTH1R*^*fl/fl*^ mutant mice. Hematoxylin and eosin (H&E)-stained sections revealed that the PDL was intact in controls, maintaining a clear separation between the root surface and the alveolar bone (Fig. 2a). In contrast, mutant molars exhibited direct fusion of alveolar bone to the root surface, indicative of ankylosis, with asterisks marking regions of abnormal bone attachment (Fig. 2b). Quantitative analysis showed that the ankylosis index in mutant mice ranged from 15% to 70%, whereas no ankylosis was observed in controls (Fig. 2c). Morphologically, low-severity cases (15%–30% fusion) displayed focal mineralized tissue bridges connecting alveolar bone to root surfaces (Fig. 2d), whereas high-severity specimens (50%-70% fusion) showed continuous mineralized tissue replacing the PDL (Fig. 2e). PTH1R staining showed PTH1R⁺ cells along the acellular cementum surface, as well as in subsets of PDL cells and on the alveolar bone surface (Fig. S3A); in *DMP1-Cre;PTH1R*^*fl/fl*^ mice, PTH1R⁺ cells were markedly reduced on the cementum surface, in the remaining PDL, and in ankylosed regions (Fig. S3B, C), confirming efficient PTH1R deletion in the mutants.

To further investigate the ankylosis phenotype and assess the integrity of the remaining PDL, we examined the expression of key PDL markers. Immunofluorescence staining for Periostin, an essential extracellular matrix protein in the PDL, revealed strong and continuous expression throughout the PDL in control mice (Fig. 2f). In contrast, *DMP1-Cre;PTH1R*^*fl/fl*^ mice displayed disrupted or completely absent Periostin expression in regions corresponding to ankylosis sites (Fig. 2g). Similarly, Decorin expression, another critical PDL matrix component, was markedly reduced in mutant mice compared to the robust expression observed in controls (Fig. 2h, i). Collagen III, a major structural component of the PDL, was abundantly expressed in control mice with well-organized fiber orientation (Fig. 2j). However, *DMP1-Cre;PTH1R*^*fl/fl*^ mice exhibited severely disorganized and markedly diminished collagen III fibers (Fig. 2k, quantified in l). Picrosirius red staining under polarized light microscopy revealed significant alterations in PDL fiber alignment in *DMP1-Cre;PTH1R*^*fl/fl*^ mutant mice. While control mice showed well-organized fibers across the PDL space (Fig. S4A), mutant mice exhibited disrupted fiber alignment even in regions where the PDL space persisted (Fig. S4B). In ankylosed areas, this staining confirmed the complete loss of PDL space and fiber structure, with direct fusion between alveolar bone and the root surface (Fig. S4C). These findings demonstrate that PTH1R signaling is critical for maintaining proper PDL fiber organization and preventing pathological ankylosis.

### Aberrant cementoblast activity leads to ankylosis in *DMP1-Cre;PTH1R*^*fl/fl*^ mutant mice

**Fig. 3:**
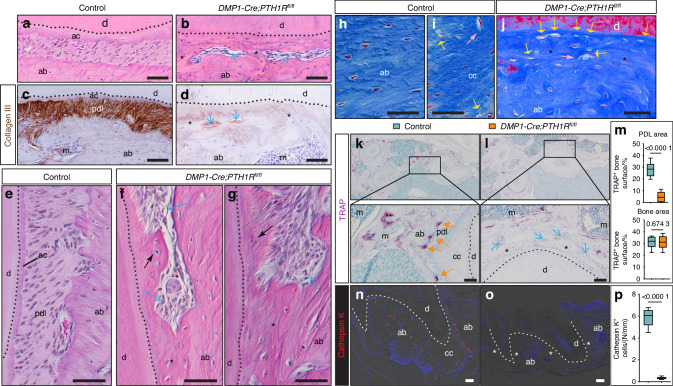
Aberrant cementoblast activity and compromised acellular cementum integrity in *DMP1-Cre;PTH1R*^*fl/fl*^ mice. **a**, **b** Representative H&E-stained sagittal sections of maxillary first molar roots. **c**, **d** Immunohistochemical staining for Collagen III to visualize the PDL. **e**–**g** H&E-stained sections showing normal and varying degrees of cementum disruption and ankylosis in mutant mice. Blue arrows indicate remaining PDL; Black arrows indicate cellular cementum located in areas typically occupied by acellular cementum. **h**–**j** Masson’s trichrome staining comparing lacunar morphology in (**h**) alveolar bone and (**i**) cellular cementum of control mice with the (**j**) ankylosed region in *DMP1-Cre;PTH1R*^*fl/fl*^ mice. Yellow arrows indicate empty lacunae; pink arrows indicate irregularly shaped lacunae. **k**, **l** TRAP staining identifies osteoclasts in transverse maxillary first molars. Higher magnification insets showing osteoclasts on the alveolar bone area in controls (orange arrows), but were absent from the remaining PDL area (blue arrows) in mutants. **m** Quantitative analysis of TRAP^+^ cells in the PDL area and marrow area (*n* = 8). **n**, **o** Immunofluorescence staining for Cathepsin K. **p** Quantification of Cathepsin K-positive cells in the PDL area (*n* = 8). Dotted lines mark the dentin boundary. Abbreviations: d, dentin; pdl, periodontal ligament; ab, alveolar bone; cc, cellular cementum; ac, acellular cementum; m, bone marrow. Asterisks indicate ankylosis regions. Data are expressed as the mean ± SD. Scale bars: 50 µm

To determine whether the ankylosis observed in *DMP1-Cre;PTH1R*^*fl/fl*^ mice resulted from aberrant cementoblast activity or alveolar bone overgrowth, we conducted detailed histological and immunohistochemical analyses. H&E-stained sagittal sections of maxillary first molar roots revealed normal periodontal architecture in control mice (Fig. 3a), while mutant mice displayed disrupted tissue organization (Fig. 3b). Based on the anatomical localization of the remaining PDL (blue arrows) in mutants, we inferred that the ectopic tissue resulted from cementum overgrowth, which displaced the PDL away from the tooth surface (Fig. 3b). Collagen III immunostaining confirmed this finding, revealing that remaining positive fibers (blue arrows) were displaced by mineralized tissue originating from the root surface (Fig. 3c, d). More specifically, analysis of mutant samples with low ankylosis index revealed cellular cementum-like tissue (black arrows) in regions normally occupied by acellular cementum, providing strong evidence for aberrant cementoblast activity (Fig. 3e–g).

Masson’s trichrome staining allowed examination of lacunar morphology in the mineralized tissues. In control mice, osteocyte lacunae within the alveolar bone were regular and ellipsoid (Fig. 3h), in stark contrast to the larger, more irregularly shaped lacunae of cementocytes in cellular cementum (Fig. 3i). The ankylosed region in mutants (Fig. 3j) contained both empty lacunae and irregularly shaped lacunae resembling cementocytes rather than osteocytes. This lacunar pattern in the ankylosed tissue provides morphological evidence for its cementum-like nature.

In control mice, TRAP-positive cells were predominantly found on alveolar bone surfaces facing the PDL, but were absent from the cementum surface (Fig. 3k). Importantly, the ankylosed regions in mutants showed a marked absence of TRAP-positive cells (Fig. 3l), suggesting that this tissue was not attracting osteoclasts as would be expected for bone tissue. Quantitative analysis confirmed a significant reduction in TRAP-positive cells in the PDL area of mutant mice compared to controls (Fig. 3m). Interestingly, there was no significant difference in TRAP-positive cell numbers in the bone marrow area between control and mutant mice (Fig. 3m), indicating that the observed phenotype is not due to a systemic impairment of osteoclastogenesis. Cathepsin K immunofluorescence staining showed a similar pattern to TRAP staining. Control mice exhibited abundant Cathepsin K-positive cells in the PDL adjacent to alveolar bone (Fig. 3n), while mutant mice displayed markedly reduced Cathepsin K expression in the PDL area (Fig. 3o). Quantification confirmed significantly fewer Cathepsin K-positive cells in the PDL area of mutant mice compared to controls (Fig. 3p).

**Fig. 4:**
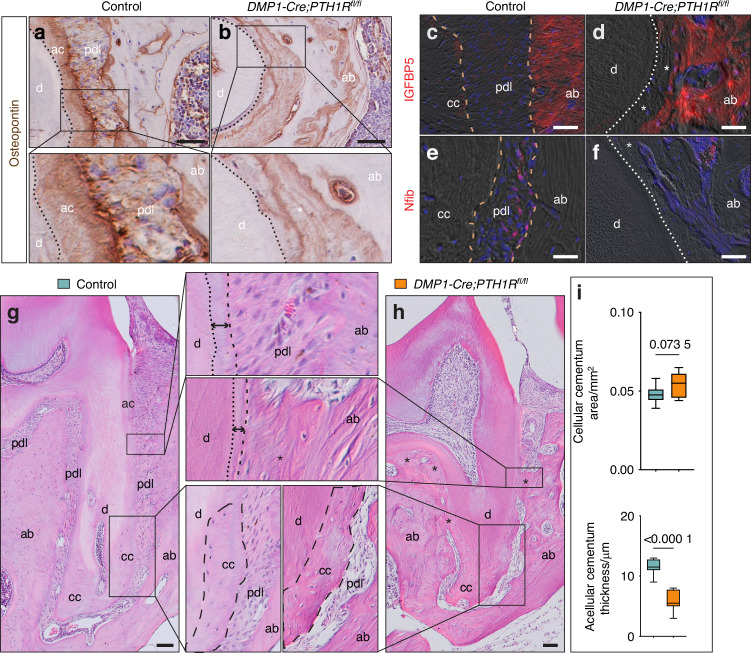
Ankylosis regions in *DMP1-Cre;PTH1R*^*fl/fl*^ mice display a cementum-like molecular identity, distinct from alveolar bone. **a**, **b** Immunohistochemical staining of osteopontin. Immunofluorescence staining for (**c**, **d**) IGFBP5 and (**e**, **f**) Nfib. **g**, **h** H&E-stained sagittal sections with corresponding higher-magnification views highlighting acellular and cellular cementum regions. **i** Quantification of acellular cementum width and cellular cementum area (*n* = 8). Data are expressed as the mean ± SD. Abbreviations: d, dentin; pdl, periodontal ligament; ab, alveolar bone; ac, acellular cementum; cc, cellular cementum. Dotted lines mark the dentin boundary. Dashed lines mark the PDL or cementum boundary. Asterisks indicate ankylosis regions. Scale bars: 50 µm

To further validate the tissue identity of the ankylosed regions at the molecular level, we examined the expression patterns of cementum- and bone-associated markers.^18–20^ Osteopontin immunostaining provided additional molecular evidence supporting this conclusion. In control mice, osteopontin expression was markedly stronger on the cementum surface than on the alveolar bone (Fig. 4a). In mutant mice, the ankylosed tissue retained this high osteopontin expression pattern (Fig. 4b), consistent with a cementum-like identity. To further characterize the lineage specificity of the ankylosed tissue, we examined several cementum-associated markers, including Jagged-1, PTHrP, Netrin-4, and CD73. All four markers were strongly expressed in ankylosed regions (Fig. S5A–H). By contrast, bone-associated markers such as insulin-like growth factor-binding protein 5 (IGFBP5) and nuclear factor I/B (Nfib) were readily detected in alveolar bone but were weak or absent in the ankylosed areas (Fig. 4c–f). Collectively, these molecular features corroborate our histological findings and support the conclusion that the ankylosed tissue is cementum-derived rather than alveolar bone.

Notably, in most affected teeth, ankylosis occurred primarily in regions normally occupied by acellular cementum, whereas areas naturally covered by cellular cementum near the apices or furcation generally preserved an intact PDL (Fig. 4g, h, Fig. S5I,J). Quantitative analysis confirmed no significant difference in cellular cementum area between the control and *DMP1-Cre;PTH1R*^*fl/fl*^ mice (Fig. 4i). The thickness of acellular cementum was reduced in the *DMP1-Cre;PTH1R*^*fl/fl*^ mutant mice (Fig. 4i). However, the presence of acellular cementum in the mutant mice (Fig. S5J) indicates that the periodontium was initially intact and that dysregulation was triggered at a later post-developmental stage rather than during early cementum formation. These findings suggest a region-specific vulnerability to pathological tissue transformation and ankylosis, with acellular cementum areas being more susceptible than those dominated by cellular cementum.

In summary, we demonstrated that ankylosis in *DMP1-Cre;PTH1R*^*fl/fl*^ mice results from cementum overgrowth rather than alveolar bone expansion. Multiple lines of evidence support this conclusion, including the remaining PDL location, ectopic cementum-like tissue, cementocyte-like lacunae, absence of osteoclast recruitment, and cementum-specific protein expression. Thus, these findings suggest that PTH1R signaling in *DMP1-Cre*-targeted cells is essential for restraining cementogenesis and maintaining periodontal homeostasis.

### PTH1R deletion leads to enhanced p-Smad3 signaling and promotes cementoblast differentiation and mineralization

**Fig. 5:**
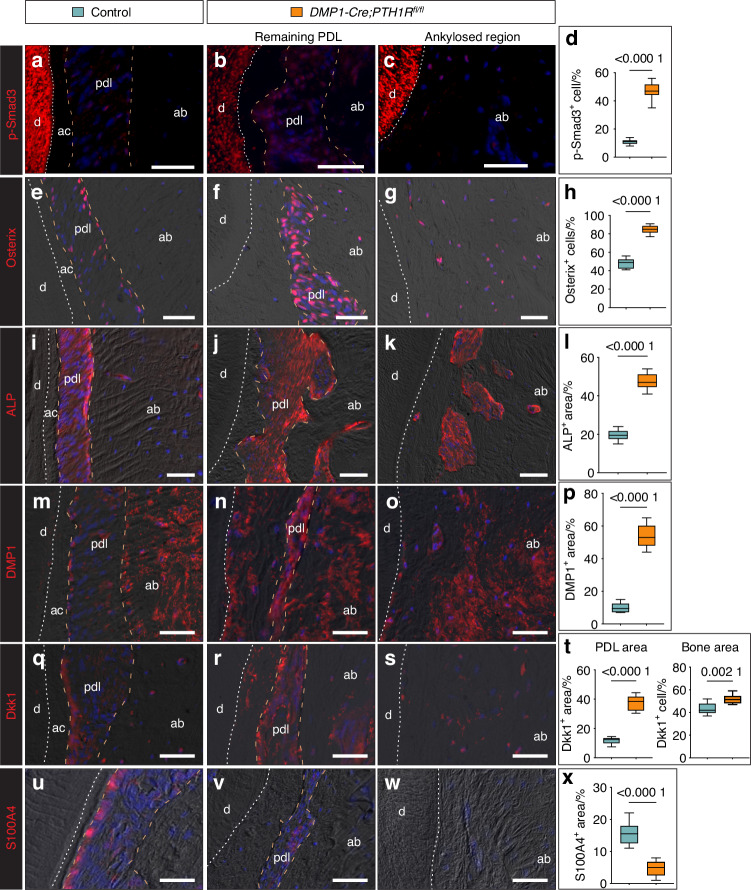
PTH1R deletion promotes cementoblast differentiation via Smad3-dependent mechanisms. **a**–**c** Immunofluorescence staining of phosphorylated Smad3 (p-Smad3). **d** Quantification of p-Smad3^+^ cells in the PDL (*n* = 8). **e**–**g** Immunofluorescence staining of Osterix. **h** Quantification of Osterix^+^ cells in the PDL (*n* = 8). **i**–**k** Immunofluorescence staining of ALP. **l** Quantification of ALP^+^ area in the PDL (*n* = 8). **m**–**o** Immunofluorescence staining of DMP1 in the PDL. **p** Quantification of DMP1^+^ area in the PDL (*n* = 8). **q**–**s** Immunofluorescence staining of Dkk1. **t** Quantification of Dkk1^+^ area in the PDL and Dkk1^+^ cells in alveolar bone (*n* = 8). **u**–**w** Immunofluorescence staining of S100A4. **x** Quantification of S100A4^+^ area in the PDL (*n* = 8). Dotted lines mark the dentin boundary. Dashed lines indicate the boundary of the PDL. Abbreviations: d, dentin; pdl, periodontal ligament; ab, alveolar bone; ac, acellular cementum. Data are expressed as the mean ± SD. Scale bars: 50 µm

To further investigate the molecular mechanisms underlying the ankylosis phenotype in *DMP1-Cre;PTH1R*^*fl/fl*^ mice, we examined key signaling pathways involved in cementoblast differentiation and mineralization. Because Sclerostin is a well-established downstream target strongly suppressed by PTH through PTH1R signaling,^21,22^ we first assessed whether Sclerostin expression was altered following PTH1R deletion. Surprisingly, Sclerostin levels in alveolar bone osteocytes showed no significant difference between *DMP1-Cre;PTH1R*^*fl/fl*^ mice and controls (Fig. S6A–C, quantified in D). Since Sclerostin remained unchanged, we turned to alternative PTH1R downstream pathways and examined TGF-β/Smad signaling. Previous studies have shown that PTH1R can form a complex with TGF-β type II receptor (TβRII), which modulates TGF-β signaling by regulating Smad3 phosphorylation.^23^ Immunofluorescence analysis revealed a significant increase in phosphorylated Smad3 (p-Smad3) positive cells in the PDL of mutant mice compared to controls (Fig. 5a–c, quantified in d). Given that Osterix, a critical transcription factor for osteoblast and cementoblast differentiation, is regulated downstream of p-Smad3 signaling,^24^ we next assessed its expression. *DMP1-Cre;PTH1R*^*fl/fl*^ mice exhibited dramatically increased Osterix^+^ cells in the PDL region (Fig. 5e–g, quantified in h). Based on adjacent-section analysis (Fig. S7), the p-Smad3^+^ and Osterix^+^ cells occupy the same anatomical domains within the remaining PDL, strongly suggesting that they represent the same activated cell population. Together, these findings indicate that PTH1R deficiency augments TGF-β/p-Smad3/Osterix signaling.

Having established the upregulation of Osterix in the PDL of mutant mice, we next examined the expression of key mineralization markers known to be directly regulated by this transcription factor. ALP, an early marker of osteoblastic and cementoblastic differentiation and a well-established downstream target of Osterix, was assessed by immunofluorescence staining. The analysis revealed markedly increased ALP^+^ area in the PDL of *DMP1-Cre;PTH1R*^*fl/fl*^ mice (Fig. 5i-k, quantified in l). Consistent with this pattern of enhanced differentiation, we also observed substantially increased expression of DMP1, a marker for mature osteoblasts and cementoblasts that is similarly regulated by Osterix, throughout the PDL of mutant mice (Fig. 5m–o, quantified in p).

We also investigated the expression of Dkk1, a Wnt signaling antagonist that functions as a downstream target of Osterix.^25,26^ Immunofluorescence staining revealed increased Dkk1 expression in the PDL region of *DMP1-Cre;PTH1R*^*fl/fl*^ mice (Fig. 5q–s), with quantification confirming significantly increased Dkk1^+^ area in the PDL and more Dkk1^+^ cells in alveolar bone and cementum of mutant mice (Fig. 5t). This upregulation of Dkk1, a negative regulator of Wnt signaling, suggests that reduced Wnt activity may work in concert with the observed p-Smad3/Osterix axis to promote cementoblastic differentiation in the absence of PTH1R. S100A4, a downstream effector of Wnt signaling,^27,28^ has been reported to directly inhibit mineralization in the PDL.^29,30^ We found that S100A4 was strongly expressed in cementoblasts but was markedly reduced in the remaining PDL of *DMP1-Cre;PTH1R*^*fl/fl*^ mutants (Fig. 5u–x).

Although Sclerostin expression was not significantly altered within most regions of the periodontal ligament or adjacent alveolar bone (Fig. S6), we observed a marked increase in Sclerostin expression specifically in the basal cortical bone and the PDL region directly attached to the basal bone (Fig. S8A, B, quantified in C). This spatially confined increase in Sclerostin aligns with our findings in long bones showing that osteocytic PTH1R is required for mechanical loading-induced suppression of Sost.^31^ Without PTH1R, bone cannot adequately downregulate Sost in response to mechanical cues. Because the basal cortical bone is continuously exposed to functional loading from mastication, the loss of PTH1R-dependent mechanotransduction likely results in persistently elevated Sclerostin, reduced Wnt-driven bone formation, and the pronounced thinning of the basal bone observed in the mutants (Fig. 1).

In addition, we assessed the expression of mineralization inhibitors associated with acellular cementum regulation.^32–34^ Notably, ANK and ENPP1 expression were significantly reduced in *DMP1-Cre;PTH1R*^*fl/fl*^ mice (Fig. S9), further supporting a shift toward a pro-mineralization microenvironment that favors aberrant cementum apposition.

**Fig. 6:**
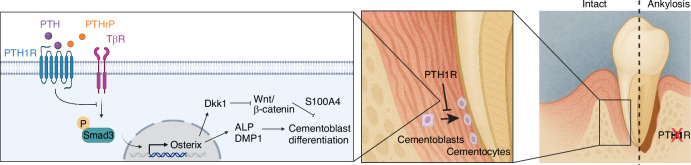
Schematic diagram illustrating the proposed mechanism underlying PTH1R-mediated regulation of cementogenesis. Under physiological conditions, PTH1R signaling in cementoblast-lineage cells restrains pro-cementogenic Smad3-Osterix activation while limiting Dkk1 expression, thereby helping to maintain adequate Wnt/β-catenin activity and balanced cementoblast differentiation. Loss of PTH1R disrupts this regulatory equilibrium, leading to Smad3 hyperactivation, elevated Osterix, and increased ALP/DMP1 expression together with upregulated Dkk1 and suppressed Wnt signaling. This coordinated dysregulation drives aberrant cementoblast activity and excessive cementum deposition, ultimately resulting in pathological root-bone fusion (ankylosis)

Collectively, these analyses reveal a molecular mechanism whereby PTH1R signaling regulates cementoblast differentiation and PDL homeostasis (Fig. 6). In physiological conditions, PTH1R signaling restrains the TGF-β/p-Smad3/Osterix axis to limit excessive cementoblastic differentiation. Concurrently, it maintains canonical Wnt signaling by suppressing the expression of its antagonist, Dkk1. The disruption of this regulatory network in *DMP1-Cre;PTH1R*^*fl/fl*^ mice therefore unleashes a dual-pronged pathogenic mechanism: it simultaneously enhances the pro-cementogenic TGF-β/p-Smad3/Osterix axis while suppressing Wnt signaling via Dkk1 upregulation. This combined dysregulation creates a potent pro-mineralization environment, culminating in excessive cementoblast differentiation and the observed ankylosis phenotype.

## Discussion

Here, we identify PTH1R signaling in cells targeted by the DMP1-Cre as a critical regulator of periodontal homeostasis. We demonstrate that its conditional deletion unleashes pathological cementum overgrowth, culminating in molar ankylosis, driven by the dysregulation of pro-cementogenic p-Smad3-Osterix signaling. These findings establish PTH1R as an essential molecular brake on cementogenesis, safeguarding the periodontium against pathological tooth-bone fusion.

### PTH1R signaling in cementogenesis

Our study demonstrates that conditional deletion of PTH1R using the *DMP1-Cre* model leads to significant ankylosis in adult mice. This observation aligns with, yet is distinct from, the findings of Ono et al.,^15^ who reported that PTH1R deletion in *Osterix-Cre*-expressing progenitors resulted in failed tooth eruption and profound root truncation, thereby establishing the importance of PTH1R in dental root formation. While both studies observed root shortening, a key difference is that our mutant mice were still able to achieve tooth eruption, which allowed for the subsequent development and pathological analysis of cementum and the observation of ankylosis. In contrast, the *Osterix-Cre*-mediated deletion described by Ono et al. did not reveal overt cementum alterations, likely because the mutation occurred at an earlier developmental stage, thus precluding normal tooth eruption, root formation and subsequent cementum maturation. The differences between these findings can be attributed to several critical methodological and biological factors. The Cre drivers used in each study target distinct cell populations and developmental windows. The *Osterix-Cre* model marks a broader population of osteoprogenitors and bone marrow stromal cells, affecting dental tissues at a much earlier stage.^35^ In contrast, the *DMP1-Cre* model used in our study targets a more restricted population, including mature osteocytes and a subset of osteoblasts,^31^ thus allowing for eruption and early cementum deposition before PTH1R deletion exerts its effects. Notably, when Ono et al. used the 10 kb *DMP1-Cre* line, the resulting mutants exhibited largely normal tooth eruption and root development, and importantly did not show root truncation or detectable cementum alterations.^15^ This discrepancy may reflect the differences in the Cre constructs: the 8 kb and 10 kb *DMP1-Cre* drivers differ in their regulatory elements and activation patterns, which might lead to differences in the specific cell populations and developmental stages in which PTH1R is deleted.^36,37^ Furthermore, the age at which PTH1R deletion was examined differs markedly between studies. Ono et al. evaluated their 10 kb *DMP1-Cre* mutants at postnatal day 25 (P25), whereas our analysis was performed in mature adult mice. Because cementum maturation, PDL space establishment, and periodontal homeostasis continue well beyond early postnatal stages, phenotypes that arise from impaired adult homeostasis may not yet be apparent at P25. It is therefore possible that similar cementum or PDL abnormalities would emerge in the 10 kb *DMP1-Cre* model if examined at later, more mature stages, consistent with our findings in adult mice.

In summary, the divergent phenotypes observed following PTH1R deletion using different Cre drivers and at different developmental stages highlight the context-dependent and stage-specific functions of PTH1R in craniofacial development and periodontal maintenance. Our model avoids early developmental defects, enabling novel identification of progressive ankylosis in adult mice. Future studies should delineate the precise spatiotemporal onset of ankylosis and test interventions targeting mineralization regulators during early pathological remodeling to preserve periodontal integrity.

### Distinct developmental origins and differential PTH1R responses of acellular and cellular cementum

The observation that ankylosis in our model predominantly affects regions of acellular cementum points to distinct regulatory mechanisms governing acellular versus cellular cementum, potentially involving differential PTH1R signaling responses. This concept of distinct regulation is underscored by fundamental differences in their developmental origins. Lineage tracing experiments by Takahashi et al.^16^ demonstrated that PTHrP-positive dental follicle cells, which are crucial progenitors in the periodontium, differentiate into cementoblasts responsible for acellular cementum formation but do not contribute to the cementoblasts forming cellular cementum near the apices or furcation. This establishes that the two cementoblast populations arise from distinct progenitor pools rather than from migratory interchange.

Further, Takahashi et al.^16^ found that PTH1R deletion within the acellular cementoblast lineage causes these cells to undergo dysregulated differentiation, producing ectopic cellular-cementum-like matrix in regions normally occupied by acellular cementum. This phenotype closely resembles that observed in our *DMP1-Cre; PTH1R*^*fl/fl*^ mice, although the cellular cementum accumulation was less pronounced in *PTHrP-CreER; PTH1R*^*fl/fl*^ mice. Together, these findings support the interpretation that the cellular-like matrix observed in our mutants results from identity disruption within the acellular cementoblast lineage, rather than from upward migration of cellular cementoblasts.

Acellular and cellular cementum differ in their developmental origins as well as in their dependence on inorganic pyrophosphate (PPi)-regulated mineralization. Acellular cementum forms slowly and is highly sensitive to local PPi availability, whereas cellular cementum mineralizes more rapidly and is largely PPi-independent.^33^ In our model, both ENPP1 and ANK, key PPi-generating regulators, were reduced following PTH1R deletion, a change expected to lower PPi and preferentially accelerate mineralization in acellular cementum regions. Notably, intermittent PTH administration has been reported to increase ENPP1 expression in bone,^38^ suggesting that intact PTH-PTH1R signaling normally helps maintain PPi-regulating enzymes. Together, these observations provide a coherent explanation for why cementum overgrowth and ankylosis in our mutants predominantly arise within acellular cementum domains.

In summary, the distinct developmental origins, formation dynamics, and differential sensitivity to PTH1R signaling strongly suggest that acellular cementum formation relies on a dedicated regulatory program that requires PTH1R input, in contrast to the more rapidly deposited cellular cementum. Building on prior studies and our new mechanistic data, we propose that PTH1R acts as a molecular “brake” during acellular cementum development by simultaneously suppressing the Smad3-Osterix cementogenic pathway and maintaining Wnt signaling, thereby ensuring controlled and orderly matrix deposition. When PTH1R is lost, this coordinated restraint is removed, leading to hyperactivation of Smad3-Osterix signaling together with reduced Wnt activity, and both changes promote excessive and dysregulated deposition of acellular cementum matrix. Cellular cementum, which forms more rapidly and appears intrinsically less dependent on this PTH1R-regulated program, remains relatively unaffected in the mutants.

### Clinical implications

Ankylosis of the permanent teeth, while presenting significant functional and esthetic challenges, represents only one manifestation within the broader spectrum of eruption disorders commonly associated with congenital anomalies. This study’s characterization of PTH1R-associated craniofacial and periodontal phenotypes provides important foundational insights that may inform future investigations into tooth eruption disorders. By elucidating the biological and molecular pathways that impede normal eruption, this research lays the groundwork for a reverse-engineering approach to better understand the physiological processes that drive successful tooth eruption. Such an understanding could fundamentally shift the clinical management of eruption disorders and has the potential to advance both conservative treatment modalities and early intervention protocols. Ultimately, these findings contribute to a deeper comprehension of eruption biology and may help resolve the long-standing question of why teeth erupt.

### Limitations and future directions

This study identifies key morphological and molecular abnormalities associated with PTH1R loss in craniofacial and periodontal tissues, yet several limitations remain. A key limitation of the present model is that the constitutive *DMP1-Cre* system precludes lineage-tracing approaches, preventing definitive determination of the cellular origin of the newly emerged DMP1^+^/ALP^+^ populations. In addition, the analysis was limited to a single developmental stage, preventing resolution of the temporal sequence through which ankylosis emerges. The absence of functional rescue experiments further limits causal inference regarding downstream signaling pathways. Future work incorporating inducible models, developmental time-course analyses, and targeted pathway perturbations will be essential to define the critical windows and molecular mechanisms driving PTH1R-dependent periodontal homeostasis and ankylosis.

## Conclusion

In conclusion, our study demonstrates that conditional deletion of PTH1R in *DMP1-Cre* targeted cells disrupts periodontium homeostasis, leading to pathological cementum overgrowth and ankylosis. These findings provide direct evidence that PTH1R signaling is required for maintaining periodontal integrity and for preventing abnormal cementum deposition and tooth-bone fusion, by maintaining the proper Smad3 and Wnt signaling network. This work advances our understanding of the molecular mechanisms underlying periodontal tissue maintenance and may inform future therapeutic strategies for preventing or treating ankylosis and related craniofacial disorders.

## Materials and methods

### Animals

In this study, we employed the 8 kb *DMP1-Cre* transgenic mouse model, which offers enhanced specificity for osteocytes compared to the 10 kb variant.^31^ While both constructs exhibit some non-specific expression in osteoblasts, muscle, and certain soft tissues, the 8 kb *DMP1-Cre* demonstrates lower expression ratios and fluorescence intensity in bone surface cells.^35,39^ Our previous work established that this Cre line targets alveolar bone osteocytes and cementocytes.^40^ Within the PDL, this Cre line targets some cells on the cementum surface and a few cells on the alveolar bone surface.^40^ To investigate the role of PTH1R in DMP1-expressing cells, we generated *DMP1-Cre;PTH1R*^*fl/fl*^ mice by crossing *DMP1-Cre* mice with *PTH1R*^*fl/fl*^ mice.^31^ 28-week-old *DMP1-Cre;PTH1R*^*fl/fl*^ and littermate *PTH1R*^*fl/fl*^ mice (hereafter referred to as control mice) were examined. All animal procedures were approved by the Institutional Animal Care and Use Committee (IACUC) of the University of Arkansas for Medical Sciences, and animal care followed institutional guidelines.

### Micro-computed tomographic (μCT) analyses

To assess bone morphology, mouse head specimens were scanned and reconstructed using our previously described method.^41,42^ Three-dimensional reconstruction was performed with CTvox software (Bruker, Billerica, MA) and subsequent measurements were taken with CTAn software (Bruker). Root length and alveolar height were measured as previously described.^43^ Basal bone thickness was assessed beneath the distal roots of the maxillary first molar in the coronal sections. Craniofacial measurements were performed using previously defined anatomical landmarks.^44^ Dentin thickness was measured as the distance between the dentinoenamel junction and the pulp chamber boundary in standardized coronal sections, as previously described.^43^

### Sample preparation

Samples underwent decalcification in 0.5 mol/L EDTA (pH 7.2) for 3–5 days. Following decalcification, tissues were dehydrated, cleared in xylene, and infiltrated with a xylene-paraffin mixture prior to paraffin embedding. Sagittal and transverse sections were cut to a thickness of 6 μm and collected on positively charged slides for analysis.

### Hematoxylin and eosin (H&E) stain

To visualize general tissue morphology, H&E staining was performed using the protocol previously described.^45^ Briefly, tissue sections were deparaffinized, rehydrated, stained with hematoxylin, differentiated with acid alcohol, counterstained with eosin Y, dehydrated through graded ethanol, cleared with xylene, and mounted using Permount medium.

### Tartrate-resistant acid phosphatase (TRAP) staining

To identify osteoclasts, TRAP staining was performed as previously described.^46^ The staining was counterstained with 0.5% Methyl Green (7114-03-06, Electron Microscopy Sciences, Hatfield, PA) for 15 s. The slides were then dehydrated in a series of ethanol and xylene and mounted with Permount mounting medium.

### Masson’s trichrome staining

Tissue sections were fixed in Bouin’s solution (#26367-01, Electron Microscopy Sciences) at 56 °C for 1 h. After washing, sections were stained with Weigert’s Iron Hematoxylin (equal parts #26367-02 and #26367-03, Electron Microscopy Sciences) for 30 min, followed by Biebrich Scarlet-Acid Fuchsin (#26367-04, Electron Microscopy Sciences) for 3 min. Sections were then treated with Phosphomolybdic-Phosphotungstic Acid solution (#26367-05, Electron Microscopy Sciences) for 25 min and Aniline Blue (#26367-06, Electron Microscopy Sciences) for 1 min. After differentiation in 1% acetic acid, sections were dehydrated through graded alcohols, cleared in xylene, and mounted with Permount. This staining protocol results in black nuclei; red cytoplasm, keratin, and muscle fibers; and blue collagen and mucin.

### Picrosirius red stain

The slides were stained with 0.1% Sirius Red in saturated picric acid (26357-02, Electron Microscopy Sciences) and visualized under polarized light using our previously described method.^47^

### Immunohistochemistry

To investigate the expression of specific proteins, immunohistochemistry staining was performed as described previously^48^ using the following primary antibodies: anti-Periostin (19899-1, Proteintech, Wuhan, China), anti-RUNX2 (ab192256, Abcam, Boston, USA), anti-Cathepsin K (ab300569, Abcam), anti-Osterix (ab22552, Abcam), anti-PTH1R (PA5-80955, Invitrogen), anti-Collagen III (22734-1-AP, Proteintech), anti-Decorin (ab277636, Abcam), anti-alkaline phosphatase (ALP, AF2910-SP, R&D Systems, Minneapolis, MN), anti-IGFBP5 (AF578, R&D Systems), anti-Netrin-4 (AF1132, R&D Systems), anti-Nfib (NBP3-32647, Novus Biologicals, Littleton, CO), anti-Jagged-1 (NB600-1161, Novus Biologicals), anti-S100A4 (810101, BioLegend, San Diego, CA), anti-CD73 (A25914, ABclonal Technology, Woburn, MA), anti-PTHrP (A3183, ABclonal Technology), and anti-Osteocalcin (M173, Takara Bio Inc, Otsu, Japan). For fluorescence detection, goat anti-rabbit IgG (H + L) cross-adsorbed antibody (A21244, Invitrogen, Carlsbad, CA) was used. For chromogenic detection, goat anti-rabbit IgG (H + L) (111-035-144, the Jackson Laboratory, Bar Harbor, ME) was used, followed by visualization with the ImmPACT DAB Substrate kit (SK4105, Vector Laboratories, Newark, CA).

### Quantification

#### All measurements were performed with Fiji (NIH, Bethesda, MD)

The ankylosis index was calculated as the percentage of the total length of the ankylosed regions relative to the total length of the roots. The formula used was ankylosis index = (ΣLength of ankylosed region / ΣRoot length) × 100.

The PDL index was calculated as the ratio of the PDL area (indicated by Collagen III-positive staining) to the tooth area in transverse sections, expressed as: PDL index = PDL area (μm²) / tooth area (μm²), where the tooth area was defined by the dentin boundary.

The TRAP^+^ surface in the transverse section was quantified by measuring the length of the TRAP^+^ surfaces relative to the total length of the alveolar bone surface. For both PDL and bone marrow areas, the percentage of TRAP^+^ surface was calculated as: TRAP^+^ surface (%) = (ΣTRAP^+^ length / Σ bone surface length) × 100.

Cathepsin K^+^ cells were quantified in sagittal sections by counting the number of positive cells along the PDL bone surface. The cell density was normalized to root length.

p-Smad3 and Osterix positive PDL cells, ALP, DMP1, Dkk1, and S100A4 positive PDL area, and Dkk1 positive osteocytes were quantified using our previously described method.^47,48^

Acellular cementum thickness was measured on H&E-stained sagittal sections of the maxillary first molar distal root adjacent to the second molar. Quantification was performed at a standardized mid-acellular-cementum location, defined as the point halfway along the acellular cementum span between the cementum-enamel junction and the transition to cellular cementum. Cellular cementum area was quantified on H&E-stained sagittal sections in the central portion of the distal root of the maxillary first molar, using matched anatomical levels across samples.^49,50^

### Statistical analysis

Results are presented as means *±* standard deviation (SD). A Student’s *t*-test was used to compare two groups. GraphPad Prism 10 (GraphPad Software, Boston, MA) was used for all statistical analyses, and significance was determined at *P* < 0.05.

## Supplementary information

Supplemental figures and figure legends
