# Supplementary material for: Loss of PTH 1 receptor signaling in periodontal cells drives cementum dysfunction and molar ankylosis in mice
Source: Bone Res. 2026 Apr 27;14:46. doi: 10.1038/s41413-026-00533-5 (PMC13111640; doi:10.1038/s41413-026-00533-5)
Supplement: Supplementary file 1 — Supplemental figures and figure legends [file 41413_2026_533_MOESM1_ESM.docx]

Supplementary Figures and Figure Legends


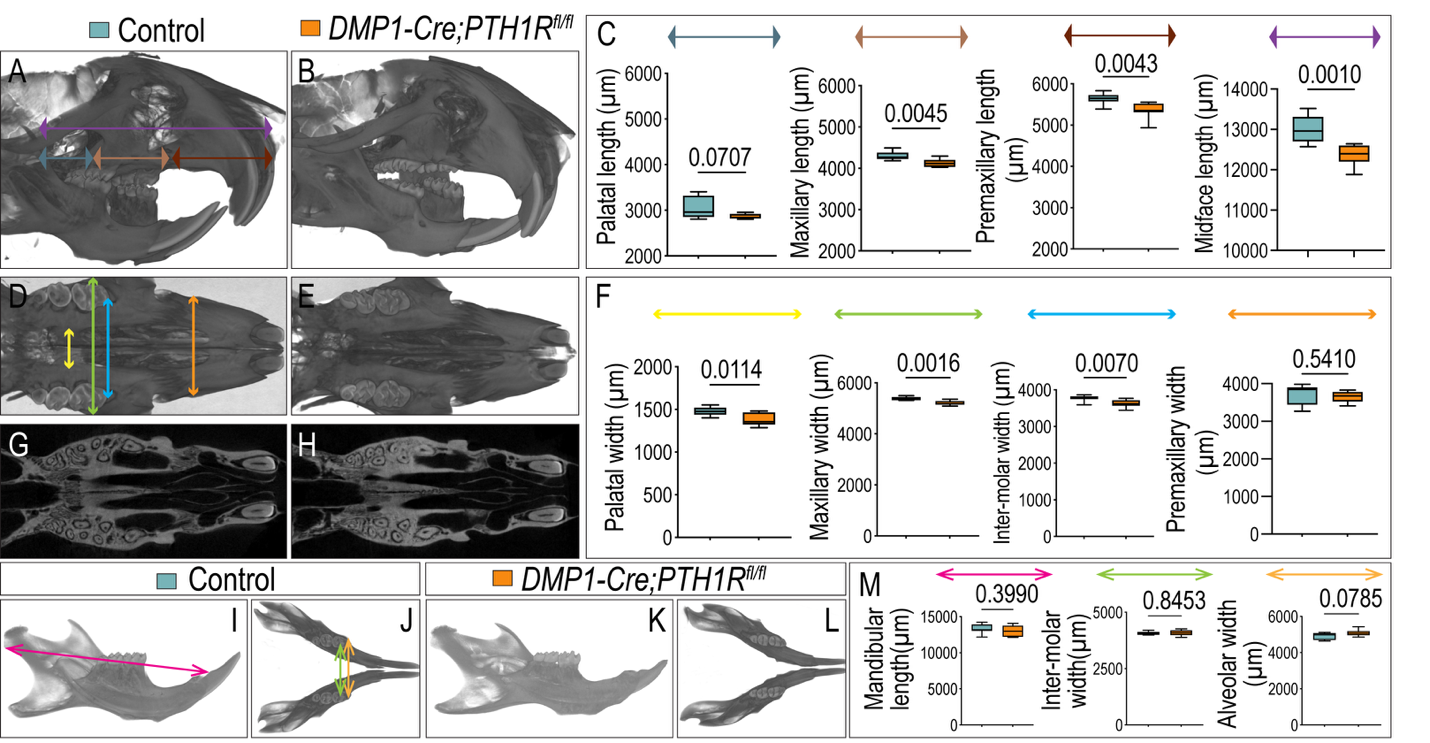


Supplementary Figure 1. Craniofacial phenotypes of *DMP1-Cre;PTH1R^fl/fl^* mutant mice.

(A,B) Representative 3D μCT reconstructions of whole skulls. (C) Quantification of craniofacial dimensions, including palatal length (blue arrow), maxillary length (orange arrow), premaxillary length (red arrow), and midface length (purple arrow) (n=8). (D,E) Representative 3D μCT reconstructions of maxillae. (F) Quantification of palatal width (yellow arrow), maxillary width (green arrow), inter-molar width (blue arrow), and premaxillary width (orange arrow) (n=8). (G,H) Transverse μCT sections of the maxillary molar region. (I-L) Representative 3D μCT reconstructions of mandibles in (I,K) sagittal and (J,L) transverse views. (M) Quantification of mandibular length (pink arrow), intermolar width (green arrow), and alveolar width (orange arrow) (n=8). Data are expressed as the mean ± SD.


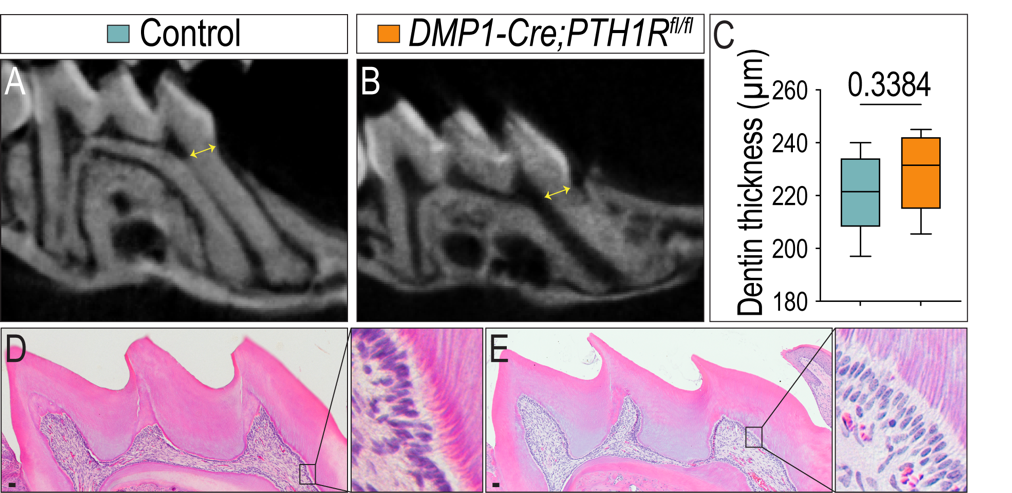


Supplementary Figure 2. Dentin analysis in *DMP1-Cre;PTH1R^fl/fl^* mice and control littermates.

(A, B) Representative μCT images of molar sections from (A) control and (B) *DMP1-Cre;PTH1R^fl/fl^* mice. Yellow arrows indicate the region where (C) dentin thickness was measured (n=8). Data are expressed as the mean ± SD. (D, E) Hematoxylin and eosin (H&E) staining of sagittal sections from (D) control and (E) *DMP1-Cre;PTH1R^fl/fl^* maxillary first molars. Scale bars: 50 μm.


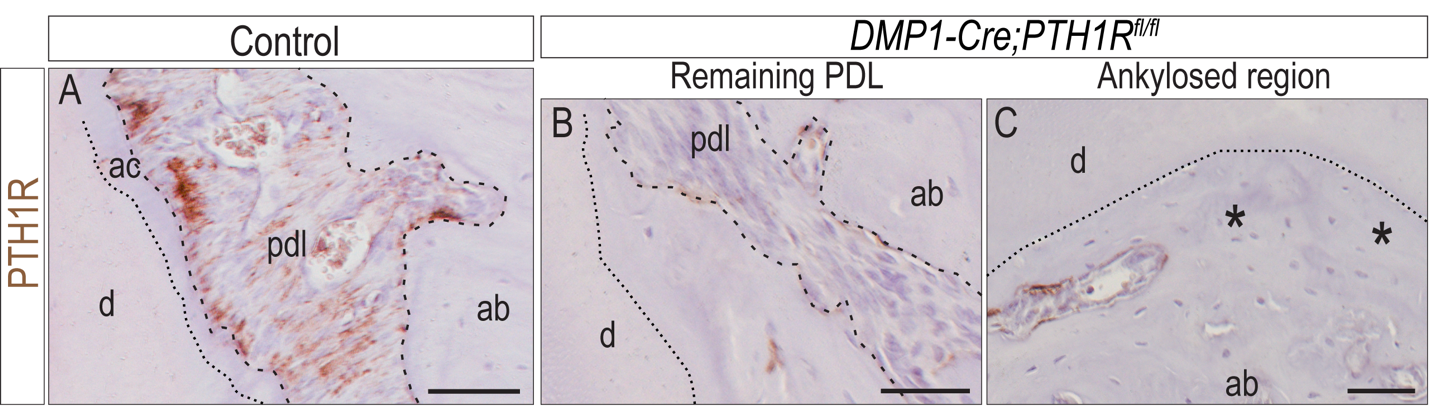


Supplementary Figure 3. PTH1R expression in control and *DMP1-Cre;PTH1R^fl/fl^* mice.

(A-C) Immunohistochemical staining for PTH1R. Abbreviations: pdl, periodontal ligament; ab, alveolar bone; d, dentin; ac, acellular cementum. Asterisks indicate ankylosis regions. Dotted lines outline the dentin boundary. Dashed lines outline the PDL boundary. Scale bars: 50 μm.


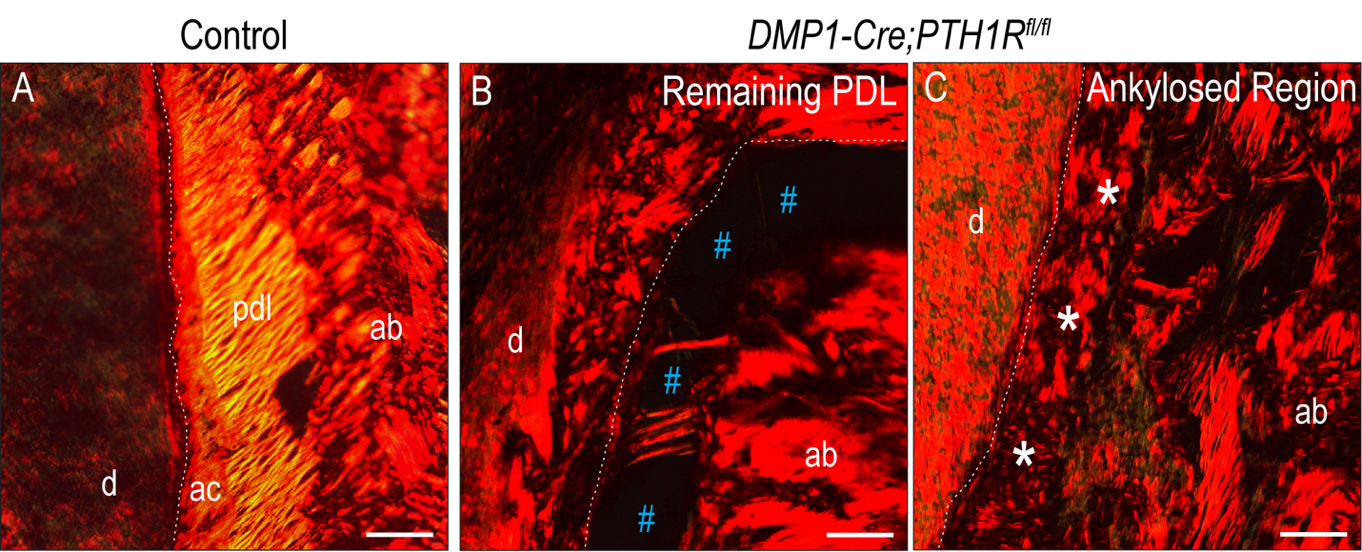


Supplementary Figure 4. Disrupted PDL and ankylosis in *DMP1-Cre;PTH1R^fl/fl^* mice.

(A-C) Picrosirius red staining of PDL fibers viewed under polarized light microscopy. Dotted lines mark the dentin boundary. Abbreviations: pdl, periodontal ligament; ab, alveolar bone; d, dentin. Asterisks indicate ankylosis regions; Hash symbols (#) indicate remaining PDL areas lacking fiber. Scale bars: 50 μm.


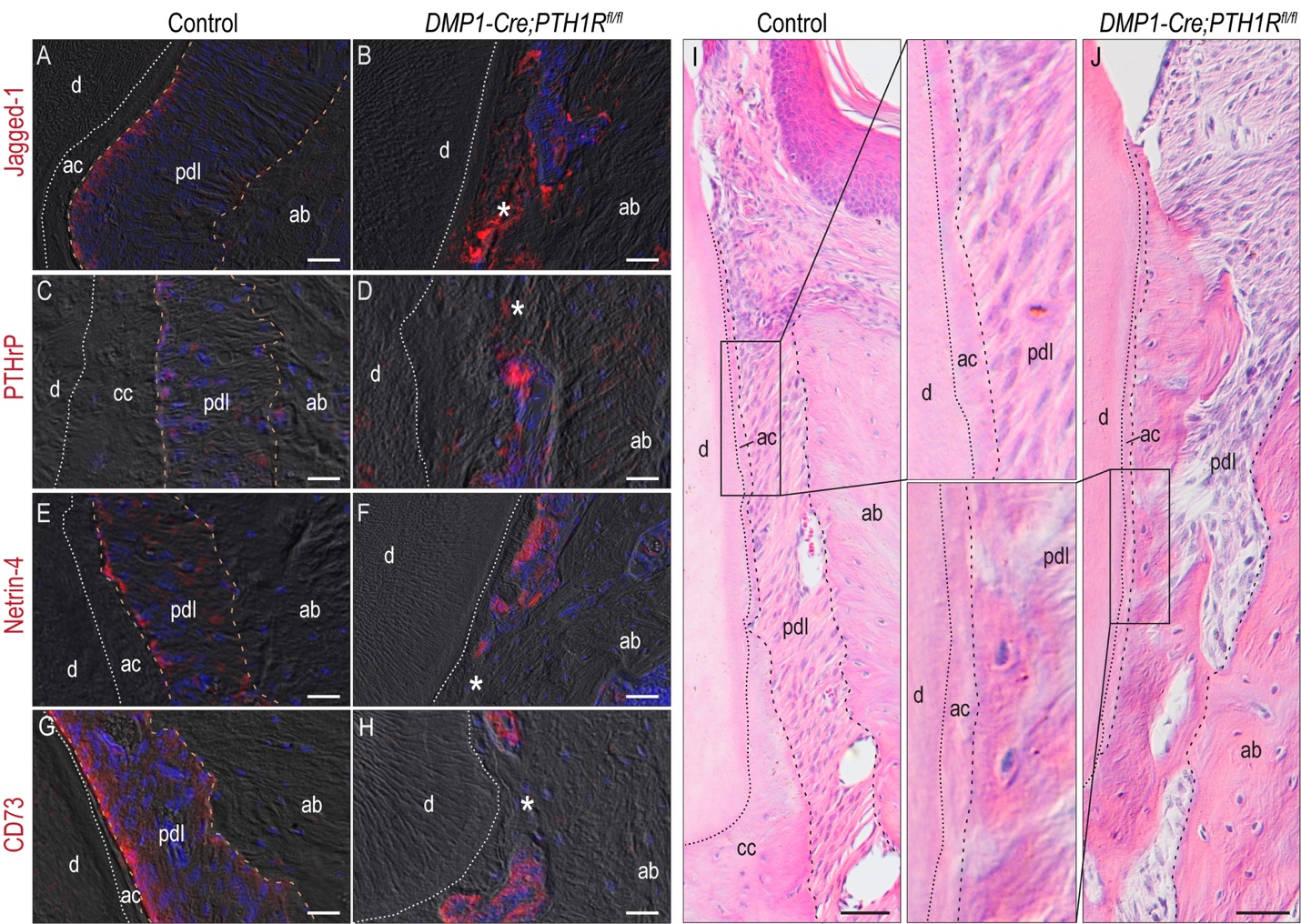


Supplementary Figure 5. Ankylosis phenotype of *DMP1-Cre;PTH1R^fl/fl^* mutant mice.

Immunofluorescence staining for (A,B) Jagged‑1, (C,D) PTHrP, (E,F) Netrin‑4, and (G,H) CD73 in maxillary first molars from control and mutant mice. (I, J) H&E-stained histological sections of maxillary first molar distal roots. Abbreviations: pdl, periodontal ligament; ab, alveolar bone; d, dentin; ac, acellular cementum; cc, cellular cementum. Dotted lines outline the dentin boundary. Dashed lines outline the PDL or cementum boundary. Asterisks indicate ankylosis regions. Scale bars: 25 μm.


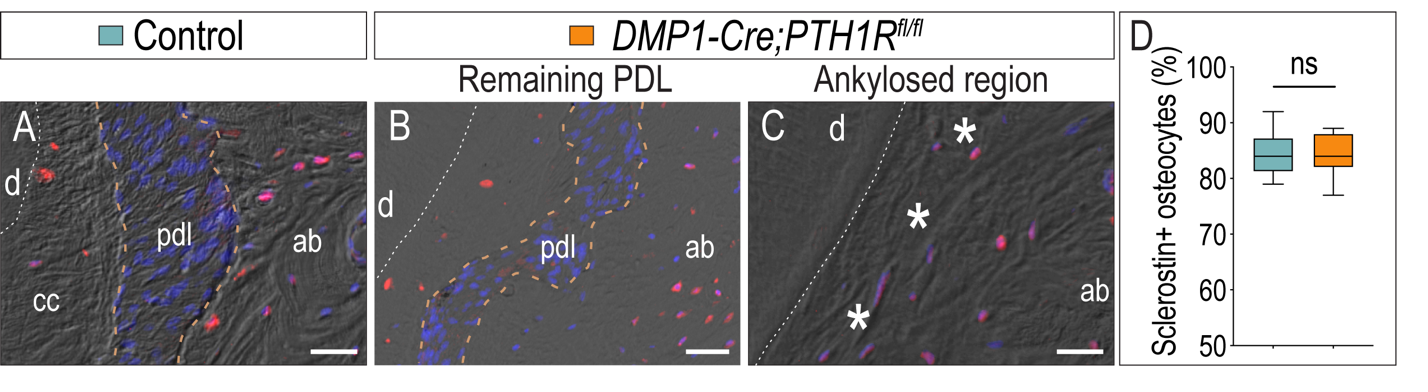


Supplementary Figure 6. Sclerostin expression following PTH1R deletion.

(A-C) Immunofluorescence staining for Sclerostin. (D) Quantification of Sclerostin-positive osteocytes in alveolar bone (n=8). Dashed lines indicate the boundary of the PDL. Dotted lines outline the dentin boundary. Asterisks indicate ankylosis regions. Abbreviations: pdl, periodontal ligament; ab, alveolar bone; d, dentin; cc, cellular cementum. Data are expressed as the mean ± SD. ns, not significant. Scale bars: 25 μm.


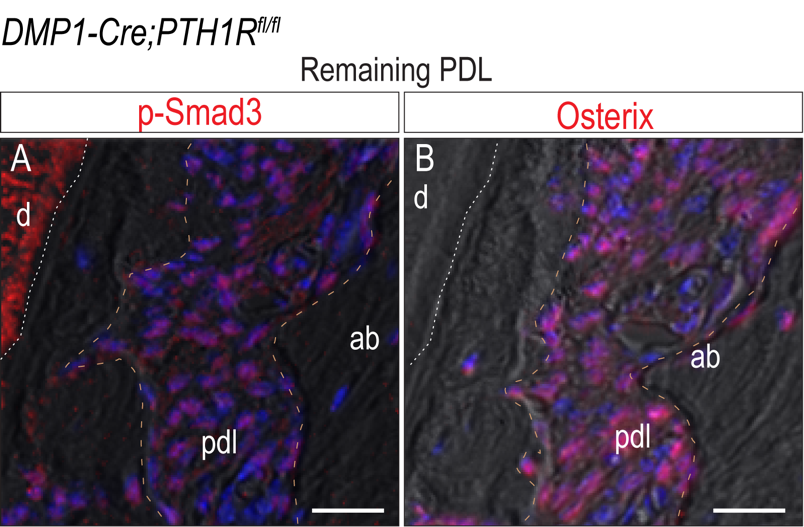


Supplementary Figure 7. Adjacent-section immunofluorescence analysis of p-Smad3 and Osterix in the remaining periodontal ligament following PTH1R deletion.

(A-B) Adjacent paraffin sections from molar periodontal tissues of *DMP1-Cre;PTH1R^fl/fl^* mice stained for (A) phosphorylated Smad3 (p-Smad3) and (B) Osterix. Dashed lines indicate the boundary of the PDL. Dotted lines outline the dentin boundary. Abbreviations: pdl, periodontal ligament; ab, alveolar bone; d, dentin. Scale bars: 25 μm.


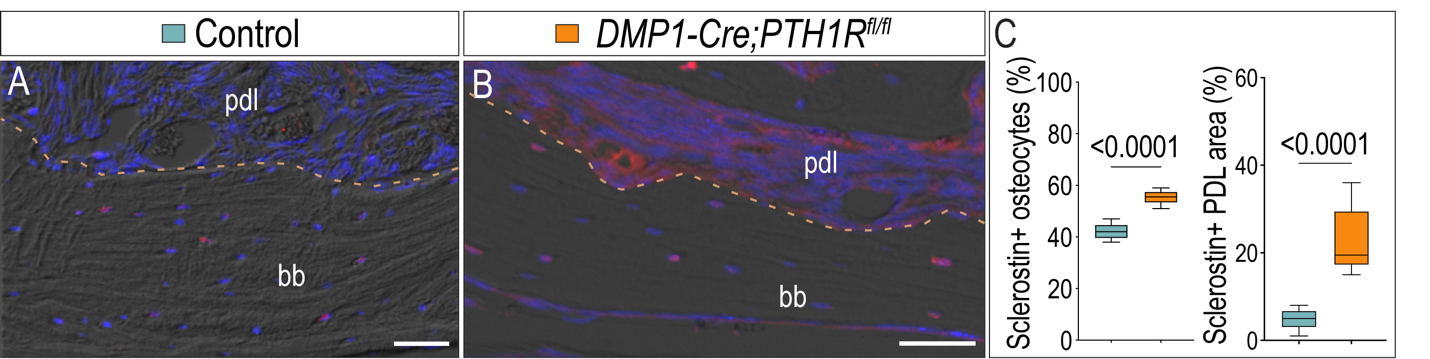


Supplementary Figure 8. Sclerostin expression in the basal bone area following PTH1R deletion.

(A,B) Immunofluorescence staining for Sclerostin in the basal bone region beneath the molar root. (C) Quantification of Sclerostin-positive osteocytes in basal bone (n=8) and Sclerostin-positive area in the basal-bone-associated PDL region (n=8). Dashed lines indicate the boundary of the PDL. Abbreviations: pdl, periodontal ligament; bb, basal bone. Data are expressed as the mean ± SD. Scale bars: 25 μm.


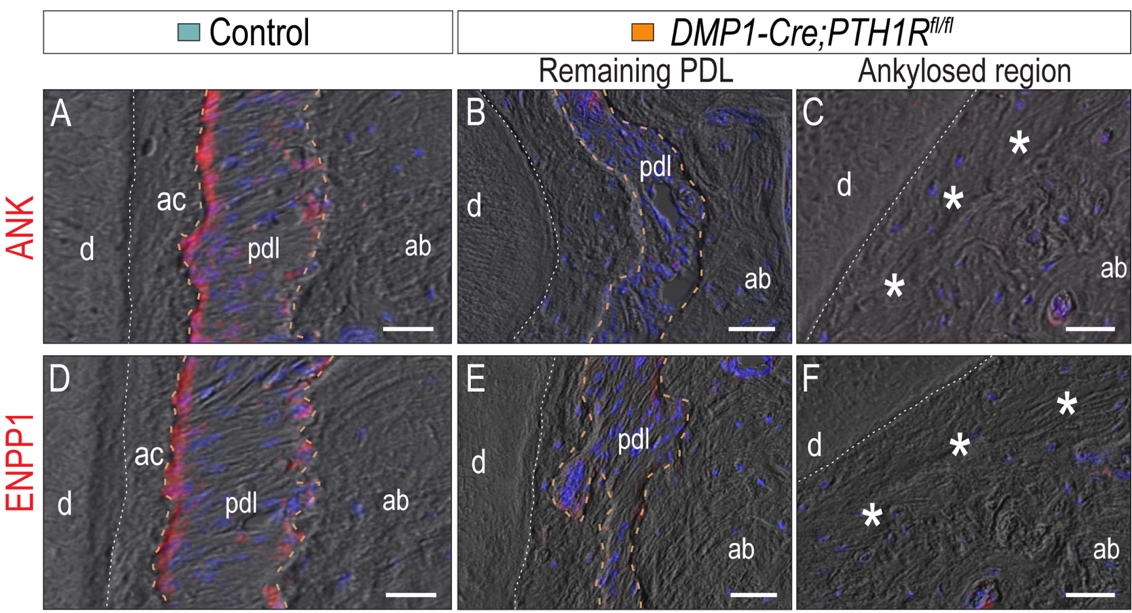


Supplementary Figure 9. Impaired extracellular pyrophosphate regulators in *DMP1-Cre;PTH1R^fl/fl^* mice.

(A-C) Immunofluorescence staining of ANK. (D-F) Immunofluorescence staining of ENPP1**.** Asterisks indicate ankylosis regions. Dotted lines outline the dentin boundary. Dashed lines indicate the boundary of the PDL. Abbreviations: pdl, periodontal ligament; ab, alveolar bone; d, dentin; ac, acellular cementum. Scale bars: 25 μm.
